# Management of uncontrolled/recurrent epistaxis by ligation or cauterization of the sphenopalatine artery: a scoping review

**DOI:** 10.1007/s00405-024-08852-1

**Published:** 2024-07-28

**Authors:** Francesco Dispenza, Francesco Lorusso, Salvatore Alberto Di Vincenzo, Anita Dolce, Angelo Immordino, Salvatore Gallina, Antonino Maniaci, Jerome Rene Lechien, Christian Calvo-Henriquez, Alberto Maria Saibene, Federico Sireci

**Affiliations:** 1 Otorhinolaringology Section, Department of Biomedicine, Neuroscience and Advanced Diagnostics, Azienda Ospedaliera Universitaria Policlinico ‘‘Paolo Giaccone’’, University of Palermo, Via del Vespro, 133, Palermo, 90127 Italy; 2 Young-Otolaryngologists of the International Federation of Oto-Rhino-Laryngological Societies (YO-IFOS) Study Group, Paris, France; 3 Faculty of Medicine and Surgery, University of Enna “Kore”, Enna, Italy; 4 Division of Laryngology and Broncho-Esophagology, Department of Otolaryngology-Head and Neck Surgery, UMONS Research Institute for Health Sciences and Technology, Mons, Belgium; 5 EpiCURA Hospital, University of Mons (UMons), Mons, Belgium; 6 Service of Otolaryngology, Hospital Complex of Santiago de Compostela, Santiago de Compostela, Spain; 7 Otolaryngology Unit, Santi Paolo E Carlo Hospital, Department of Health Sciences, Università Degli Studi di Milano, Milan, Italy

**Keywords:** Epistaxis, Sphenopalatine artery ligation, Sphenopalatine artery cauterization, Review

## Abstract

**Purpose:**

The control of epistaxis has always posed a significant challenge for otolaryngologists. One of the most viable options to address refractory cases is the ligation or cauterization of the sphenopalatine artery. The objective of this study was to assess the efficacy, safety, and long-term outcomes of these interventions.

**Materials and methods:**

Two independent otolaryngologists conducted a comprehensive search for studies dealing with management of uncontrolled/recurrent epistaxis by consulting the main scientific databases on the web, including PubMed, Google Scholar, Medline, EMBASE, Web of Science, and the Cochrane Library. The systematic review was conducted according to the Preferred Reporting Items for Systematic Reviews and Meta-analyses (PRISMA) statement. The criteria for considering studies for the review were based on the population, intervention, comparison, outcome, timing and setting (PICOTS) framework.

**Results:**

Sixteen studies were included in the systematic review, comprising a total of 454 patients. Among these, 289 individuals underwent ligation of the sphenopalatine artery, while 100 underwent cauterization of the same artery. Additionally, 56 patients underwent both ligation and cauterization of the sphenopalatine artery during the same surgery. The incidence of rebleeding and complications was respectively 12.1% (55/454) and 3% (14/454), resulting in relatively low rates in both cases.

**Conclusions:**

Our review emphasizes the increasing importance of surgical approaches, specifically ligation or cauterization of the sphenopalatine artery, in addressing refractory cases. The low incidence of complications, predominantly temporary decreased lacrimation in patients undergoing ligation of the sphenopalatine artery, highlights the safety and feasibility of these interventions.

## Introduction

The control of epistaxis has always presented a significant challenge in Otolaryngology. This nasal hemorrhagic phenomenon can arise from various causes, including trauma, local pathologies, hematological disorders, arterial high blood pressure, use of anticoagulants, and as a complication during endoscopic sinus surgery [1–3]. Despite numerous therapeutic approaches available, a subgroup of patients continues to experience epistaxis refractory to conventional therapies, requiring more advanced management strategies [4].

The branches of the sphenopalatine artery (SPA) represent the main site of epistaxis, while a lower percentage of cases involve the branches of the anterior ethmoidal artery (AEA) and the Stamm’s S-point, an arterial pedicle located in the upper septum near the projection of the axilla of the middle turbinate, posterior to the septal body [5].

One of the most valid options to address refractory epistaxis involves occluding the sphenopalatine artery (SPA), a critical branch of the internal maxillary artery, either through the application of metal clips or by cauterization using bipolar forceps. The sphenopalatine artery plays a fundamental role in supplying the nasal mucosa and surrounding structures, making it a potential site of origin and maintenance of persistent nosebleeds. Therefore, targeted SPA manipulation through ligation or cauterization procedures is a focused therapeutic approach to managing uncontrollable nasal bleeding [6].

The procedures for cauterization and ligation of the SPA are usually performed under general anesthesia with endoscopic view to achieve clear visualization of the internal nasal structures. To expose the sphenopalatine artery endoscopically, a posterolateral mucosal flap is raised over the orbital process of the palatine bone, followed by a vertical incision inferior to the posterior middle turbinate, 1 cm anterior to its posterior tip. Elevating the mucoperiosteal flap reveals the ethmoid crest, a key landmark anteromedial to the sphenopalatine foramen. Resecting the ethmoid crest enhances visibility and identification of the sphenopalatine artery and its branches. An alternative approach involves performing a middle antrostomy that reaches the posterior wall of the sinus, opening the sphenopalatine foramen and pterygopalatine space to access the SPA more proximally.

In cauterization procedures, the SPA is cauterized using an electrosurgical instrument or a radiofrequency device, which seals the artery and interrupts the blood flow. In ligation procedures, the artery is instead tied off with surgical clips to ensure hemostasis. After either cauterization or ligation, a nasal pack may be inserted, if necessary, to prevent postoperative bleeding [6].

At present, there are no prospective studies comparing the efficacy and risks of SPA ligature versus cauterization. As a consequence, the technique selection lies in the surgeon’s preference and - understandably enough - the technical instrumentation availability.

This systematic review aimed to examine in detail the efficacy, safety, and long-term outcomes associated with ligation or cauterization of the sphenopalatine artery in the treatment of intractable epistaxis. Through a critical analysis of clinical studies, systematic reviews, and related scientific research, we aim to offer a comprehensive overview of the current evidence in this area.

## Materials and methods

The current systematic review was conducted in accordance with the guidelines established by the Preferred Reporting Items for Systematic Reviews and Meta-analyses group (PRISMA), applying the PRISMA 2020 Checklist (http://www.prisma-statement.org*).*

Two independent authors conducted a comprehensive search by consulting the main scientific databases on the web, including PubMed, Google Scholar, Medline, EMBASE, Web of Science, and the Cochrane Library. Specific keyword pairs such as “epistaxis” (OR “nasal bleeding” OR “nosebleed” OR “rhinorrhagia”) AND “sphenopalatine artery” AND “management” (OR “therapy” OR “treatment” OR “surgery”) were used for the search. Titles and abstracts were reviewed to screen out non-relevant articles and the working group reviewed the full text of remaining articles. The results of the studies were then combined, integrated, and analyzed.

The criteria for considering studies for the review were based on the population, intervention, comparison, outcome, timing and settings (PICOTS) framework.

### Population and inclusion criteria

Studies in English language on patients of all ages, genders, and ethnicities with uncontrolled/recurrent epistaxis.

### Intervention

Studies in which patients underwent SPA ligation and/or cauterization after spontaneous or post-traumatic epistaxis.

### Comparison and outcome

The primary outcomes assessed were rebleeding, rebleeding time, rebleeding treatment, and the occurrence of complications, making a comparison between results obtained with SPA ligation and cauterization.

### Timing

Studies published up to December 2023 have been included in this literature review.

### Setting

Randomized controlled trials (RCT), non-randomized controlled trials (NRCT), prospective or retrospective cohort studies and case-control studies from community, private and tertiary care university hospitals were included.

## Results

**Fig. 1:**
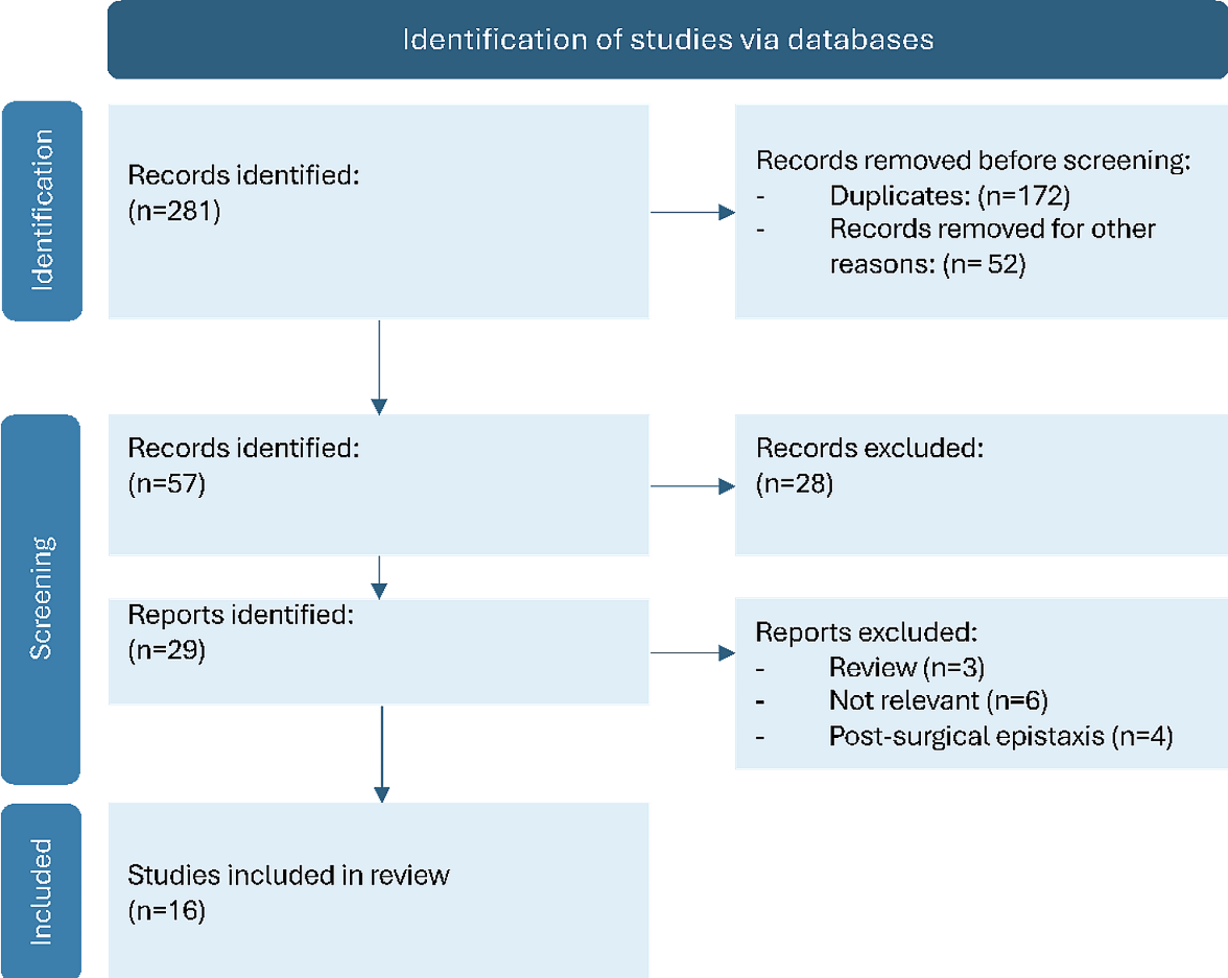
PRISMA 2020 flow diagram

A total of 281 articles were identified. A first screening allowed us to eliminate 172 duplicates, and therefore, to consider the remaining 109 articles. Fifty-two articles were excluded based on title/abstract screening, allowing us to select 57 articles for full-text screening. Then, the application of inclusion and exclusion criteria allowed us to select only 16 papers for inclusion in the review (Fig. 1).

In most cases, exclusion from the review was due to the types of study design, e.g. case reports, study groups including patients with post-surgical epistaxis, and studies with incomplete clinical data. Four [7–10] of the sixteen studies were prospective studies; the others were retrospective studies [11–22].

### Study groups

The number of participants in the included studies varied from 4 to 65, while the average age varied from 43.3 to 71. The mean age of patients undergoing cauterization [7, 9, 15] and ligation [10, 12, 13, 16–18] was 53.4 and 64.6 years, respectively. Table 1 presents the selected manuscripts along with the baseline characteristics of each study group.

**Table 1:** Selected manuscript and baseline characteristics of the study groups

Author	Year	Study title	Study design	*N* of cases	Mean age	Risk factors
Gandomi et al. [7]	2013	Endoscopic Cauterization of the Sphenopalatine Artery to Control Severe and Recurrent Posterior Epistaxis	Prospective	27	45.3	None
Abdelkade et al. [8]	2007	Endoscopic control of the sphenopalatine artery for epistaxis: long-term results	Prospective	43	68.5	NS
Wiorowski et al. [9]	2003	Indications and results of cauterization by endoscopic approach of the sphenopalatine artery in severe posterior epistaxis	Prospective	10	66.4	Antiplatelet/Anticoagulants (2) CVD (5)
O’Flynn and Shadaba [10]	2000	Management of posterior epistaxis by endoscopic clipping of the sphenopalatine artery	Prospective	12	59	Antiplatelet/Anticoagulants (1) CVD (6) Cocaine abuser (1) Wegener (1)
Seno et al. [11]	2009	Endoscopic ligation of the sphenopalatine artery and the maxillary artery for the treatment of intractable posterior epistaxis	Retrospective	8	59.9	Antiplatelet/Anticoagulants (NA) Hereditary hemorrhagic telangiectasia (1)
Bhaskar et al. [12]	2000	Endoscopic endonasal ligation of the sphenopalatine artery	Retrospective	6	59.5	Hereditary hemorrhagic telangiectasia (1)
Wormald et al. [13]	2000	Endoscopic Ligation of the Sphenopalatine Artery for Refractory Posterior Epistaxis	Retrospective	13	55.9	Antiplatelet/Anticoagulants (8) CVD (9)
Thakar and Sharan [14]	2005	Endoscopic sphenopalatine artery ligation for refractory posterior epistaxis	Retrospective	4	45	Alcohol abuse (1)
Srinivasan et al. [15]	2000	Surgical management of intractable epistaxis: audit of results	Retrospective	10	62.4	Antiplatelet/Anticoagulants (3)
de Bonnecaze et al. [16]	2018	Transnasal Endoscopic Sphenopalatine Artery Ligation Compared With Embolization for Intractable Epistaxis: A Long-term Analysis	Retrospective	39	70	Antiplatelet/Anticoagulants (15) Coagulopathy (3) Hereditary hemorrhagic telangiectasia (2)
Snyderman et al. [17]	1999	Endoscopic Sphenopalatine Artery Ligation Is an Effective Method of Treatment for Posterior Epistaxis	Retrospective	38	65	Antiplatelet/Anticoagulants + CVD (1) Coagulopathy (1)
Asanau et al. [18]	2009	Sphenopalatine and Anterior Ethmoidal Artery Ligation for Severe Epistaxis	Retrospective	45	71	Antiplatelet/Anticoagulants (34) CVD (27)
Gede et al. [19]	2013	National long-lasting effect of endonasal endoscopic sphenopalatine artery clipping for epistaxis	Retrospective	42	61.2	Antiplatelet/Anticoagulants (34) CVD (22) Hepatopaty (1) Alcohol abuse (16)
Umapathy et al. [20]	2005	Persistent epistaxis: what is the best practice?	Retrospective	41	61	Antiplatelet/Anticoagulants (9) CVD (7)
Hey et al. [21]	2019	Endoscopic Sphenopalatine Artery Ligation: General Applicability in a Teaching Unit	Retrospective	65	58.2	NS
McDermott et al. [22]	2015	Sphenopalatine Artery Ligation for Epistaxis: Factors Influencing Outcome and Impact of Timing of Surgery	Retrospective	45	62.6	Antiplatelet/Anticoagulants (19) CVD (18) Diabetes (8)

NA = not available, NS = not specified, CVD = cardiovascular diseases

In all the studies we analyzed, only patients who underwent ligation and/or cauterization of the sphenopalatine artery were included. In three studies [7, 9, 15], patients underwent only cauterization, while in eight studies [10–13,16−18], surgeons used only ligation of the sphenopalatine artery; one study [14] relied on a combination of both techniques.

Only in the study conducted by Gandomi [7] were patients with any risk factors for epistaxis excluded, while in two studies [8, 21], risk factors were not specified. In all other studies, patients had different risk factors, including high blood pressure, use of antiplatelets and anticoagulants, alcohol and cocaine abuse, liver disease, hereditary hemorrhagic telangiectasia and Wegener’s disease.

Among patients undergoing cauterization [7, 9, 15], 4.2% (2/47) had a post-traumatic etiology, 95.8% (45/47) had spontaneous epistaxis; 10.6% (5/47) had cardiovascular risk factors, and 10.6% (5/47) were on antiplatelet or anticoagulant therapy. Among patients undergoing ligation [10, 12, 13, 16–18], 3.6% (7/194) had a post-traumatic etiology 96.4% (187/194) had spontaneous epistaxis; 35.6% (69/194) had cardiovascular risk factors; 3.6% (7/194) had a combination of cardiovascular risk factors and intake of antiplatelets or anticoagulants; 2.1% (4/194) had a coagulopathy; 1.55% (3/194) had hereditary hemorrhagic telangiectasia; 0.51% (1/194) had Wegener’s disease, and another 0.51% (1/194) had a history of cocaine abuse.

In fourteen studies [7,8,10–15,17−22], patients had spontaneous epistaxis. Two studies only included patients with post-traumatic epistaxis: Wiorowski et al. [9] reported that three patients had post-traumatic epistaxis and underwent SPA cauterization and ligature of the anterior and posterior ethmoidal arteries by external approach due to the observation of additional perioperative rebleeding. De Bonnecaze et al. [16] reported that seven patients had post-traumatic epistaxis and underwent SPA ligation and systematic embolization of the internal maxillary. In four studies [15, 17, 18, 22] reporting only spontaneous epistaxis, other arteries besides the sphenopalatine artery were treated. In six studies [9, 12–15], patients were treated only unilaterally, and only in Asanau’s work [18] patients were treated always bilaterally. The hospital stay varied from 1 to 4 days, except for Asanau’s study [18] (5.5 ± 3.3 days). Table 2 reports data regarding the surgical procedure performed, hospital stay, and follow-up.

**Table 2:** Surgical procedure, postoperative recovery, and follow-up

Author	Surgical procedure (*n*)	Unilateral/bilateral (*n*)	Procedure on different arteries	Complications	Mean postoperative recovery (days)	Mean Follow-up
Gandomi et al. [7]	Cauterization (27)	Unilateral (24) Bilateral (3)	0	None	1.6	6.2 months
Abdelkade et al. [8]	Cauterization (8) Ligation (27) Cauterization + Ligation (10)	Unilateral (41) Bilateral (2)	0	None	1.5	1.32 years
Wiorowski et al. [9]	Cauterization (10)	Unilateral (10)	4	None	2.1	NA
O’Flynn and Shadaba [10]	Ligation (12)	Unilateral (10) Bilateral (2)	0	None	1	9 months
Seno et al. [11]	Ligation (8)	Unilateral (7) Bilateral (1)	0	None	1.6	NA
Bhaskar et al. [12]	Ligation (6)	Unilateral (6)	0	None	1.6	NA
Wormald et al. [13]	Ligation (13)	Unilateral (13)	0	None	1.9	13 months
Thakar and Sharan [14]	Cauterization + Ligation (4)	Unilateral (4)	0	None	3	NA
Srinivasan et al. [15]	Cauterization (10)	Unilateral (10)	4	None	2.1	10 months
de Bonnecaze et al. [16]	Ligation (39)	Unilateral (29) Bilateral (10)	0	persistent diplopia (1) case of soft tissue necrosis (1) acute sinusitis (4)	3.6	NA
Snyderman et al. [17]	Ligation (38)	Unilateral (34) Bilateral (4)	25	numbness of the teeth, palate, or upper lip (5) septal perforation (1), acute sinusitis (1), decreased lacrimation (1)	3	10 months
Asanau et al. [18]	Ligation (45)	Bilateral (45)	25	decreased lacrimation (NS)	2–8	13.7 months
Gede et al. [19]	Cauterization (11) Ligation (13) Cauterization + Ligation (18)	Unilateral (42)	0	None	2.5	6.7 years
Umapathy et al. [20]	Ligation (41)	Unilateral (39) Bilateral (2)	0	None	4	3.6 years
Hey et al. [21]	Cauterization (26) Ligation (16) Cauterization + Ligation (18) Unknown (5)	Unilateral (63) Bilateral (2)	0	None	NA	1–8 years
McDermott et al. [22]	Cauterization (8) Ligation (31) Cauterization + Ligation (6)	Unilateral (44) Bilateral (1)	1	None	1	NA

NA = not available, NS = not specified

### Outcomes

Of the total patients treated, 12,1% (55/454) relapsed after a variable time. Only in the study conducted by Thakar et al. [14], there were no cases of rebleeding. In all other studies, rebleeding underwent conservative treatment (including local cautery and nasal packing), reintervention, or was left untreated. In all studies, the time of rebleeding is reported, except for McDermott et al.’s study [22], so it is possible to distinguish between early (< 30 days) and late (> 30 days) rebleeding [23]. The rebleeding rate for patients who underwent cauterization [7, 9, 15] was 12.7% (6/47) compared to 13.8% (28/202) for those who underwent ligation [10–13,16−18,20] (*p* = 0.352, α = 0.05). Data regarding rebleeding and its treatment are presented in Table 3.

**Table 3:** Rebleeding cases and treatment

Author	Rebleeding cases	< 30 days	> 30 days	Treatment
Gandomi et al. [7]	4/27 (14.8%)	3	1	- Conservative management including local cautery and anterior nasal packing
Abdelkade et al. [8]	6/43 (13.9%)	4	2	-Endoscopic diathermy of the bleeding sites and septoplasty (2) -Conservative management (2) -None (2)
Wiorowski M et al. [9]	1/10 (10%)	1	0	**-**Embolization (1)
O’Flynn and Shadaba [10]	2/12 (1.7%)	1	1	**-**None
Seno et al. [11]	1/8 (12.5%)	0	1	**-**Packing (1)
Bhaskar et al. [12]	1/6 (16.7%)	1	0	**-** Local cautery (1)
Wormald et al. [13]	1/13 (7.7%)	0	1	- Conservative management including local cautery and anterior nasal packing
Thakar and Sharan [14]	0/4 (0%)	0	0	-
Srinivasan et al. [15]	1/10 (10%)	1	0	**-** Packing (1)
de Bonnecaze et al. [16]	10/39 (25.6%)	NS	NS	**-** Packing (10)
Snyderman et al. [17]	5/38 (13.1%)	4	1	- Reintervention (2) - Packing (3)
Asanau et al. [18]	7/45 (15.6%)	NS	NS	- Reintervention (1)
Gede et al. [19]	4/42 (10%)	3	1	- Packing (5) - Reintervention (4)
Umapathy et al. [20]	1/41 (2.4%)	0	1	- Reintervention (1)
Hey et al. [21]	5/65 (7.7%)	5	0	- Clip and diathermy of AEA and PEA (1) - Embolization (1) - Packing (2) - Reintervention ESPAL (1)
McDermott et al. [22]	6/45 (13.3%)	NS	NS	- Packing (4) - Reintervention (2)

NS = not specified, AEA = anterior ethmoidal artery, PEA = posterior ethmoidal artery, ESPAL = endoscopic sphenopalatine artery ligation

Complications were reported in only three studies [16–18], all involving patients who had undergone the ligation of the SPA (Table 2). Snyderman et al. [17] reported five cases of numbness of the teeth, palate, or upper lip, one case of septal perforation, one case of acute sinusitis, and one case of temporary decreased lacrimation. Asanau et al. [18] reported temporary decreased lacrimation in nearly all of the 45 cases. In his study, de Bonnecaze [16] reported one case of persistent diplopia due to superior oblique muscle injury. In the other studies, no complications were reported.

Epistaxis represents one of the most common emergencies treated in otolaryngology field. Whether it is traumatic, post-surgical, or spontaneous, the clinician’s aim is always to stop the nosebleed as soon as possible and with the least patient discomfort, and subsequently treat the precipitating condition [1]. Spontaneous nosebleeds are the most common, especially in elderly populations or those with one or more risk factors [5]. From our review, it emerged that the weighted average age of patients affected by spontaneous epistaxis is 62.17 years, confirming that this pathology more consistently involves the over-60 population groups.

Regarding the comorbidities presented by the patients examined, although several studies refer sparsely to conditions favoring epistaxis, it is not possible to extract the data relating to their incidence in a precise and statistically useful manner. However, as can easily be understood, the most common risk factors are represented by cardiovascular diseases (primarily arterial high blood pressure) and concomitant antiplatelet/anticoagulant therapy. The higher involvement of the over-60 population could therefore be correlated with the higher incidence of cardiovascular diseases and the use of antiplatelet/anticoagulant medications in this class of patients.

Other less frequent conditions, but still reported in studies, are possible coagulopathies, vasculopathies (e.g. hereditary hemorrhagic telangiectasia), and diabetes [24].

Regardless of the etiology, the most commonly practice is to stop the nosebleed with nasal packing. Nowadays, this procedure involves the use of expandable sponges that, once introduced into the bleeding nasal cavity, most often allow the nasal bleeding to be stopped. Packing represents an effective method, especially for anterior nosebleeds, i.e., those coming from the most anterior portion of the septum (locus Valsalvae) [25, 26]. When the bleeding comes from more posterior districts of the nasal cavity, special inflatable devices can be used.

In case of failure or rebleeding surgical hemostasis can be considered. The two techniques that we considered for this study to surgically control epistaxis are cauterization and/or ligation of the sphenopalatine artery. The total number of patients belonging to the 16 studies included in the review is 454. Of these, 289 underwent ligation of the sphenopalatine artery, while 100 underwent cauterization of the same artery. In a more limited number of cases (56), both techniques were used to control bleeding. Nine studies out of 16 reported, in some cases, the concomitant treatment of another artery (most frequently the anterior ethmoidal artery) for a total of 98 patients.

From this first analysis, it can be deduced that surgeons, for reasons ranging from greater reliability to quicker execution, prefer ligation as a surgical technique. Furthermore, several studies [27, 28] have found that ligation is a more cost-effective treatment strategy compared to nasal packing or embolization. For all these reasons, ligation should be considered first line therapy for severe/refractory posterior epistaxis.

Regarding laterality, 376 patients were treated on one side only, while 72 were operated on bilaterally. Taking into consideration the studies that presented pure data on each type of treatment (cauterization or ligation) the rebleeding rate for patients who underwent cauterization [7, 9, 15] was comparable to those who underwent ligation [10–13,16−18,20] (12.7% vs. 13.8%; 6/47 vs. 28/202; *p* = 0.352, α = 0.05) therefore, no significant differences in terms of rebleeding risk between the two techniques were detected, making the efficacy of the two procedures similar. Although it is not specified how many and which patients, the most used procedures in controlling recurrences were packing, local cautery, bilateral clipping of the anterior ethmoidal artery, repositioning of the previously positioned clip, and embolization of the sphenopalatine artery.

Regarding the average hospitalization time in patients undergoing surgical control of epistaxis, only 12 studies report this parameter in detail. Based on what they reported, the weighted average of the average length of hospitalization is 1.82 days.

Our review makes it evident that the ligation and/or cauterization of the sphenopalatine artery is a treatment with a very low incidence of complications, which were reported exclusively in 3 out of 16 studies [16–18]. In all three studies, complications resulted from the ligation of the SPA. Two studies [16, 17] only reported the exact incidence of complications, present in a total of 3% of patients (14/454) and included numbness of the teeth, palate, or upper lip, septal perforation, acute sinusitis, diplopia, and temporary decreased lacrimation. The last one represents the most frequent complication, and it was found in nearly all of the 45 cases described by Asanau et al. [18].

However, such complications might be secondary to prior failed management, such as extensive cautery and/or prolonged packing, rather than surgical ligation, therefore, we believe both procedures exhibit a similar level of safety.

The current investigations in the field of Otolaryngology, while commendable, come with certain limitations that warrant thoughtful consideration. One primary limitation arises from the decision to exclusively include studies conducted in English, potentially excluding relevant research in other languages and constraining the generalizability of findings. The incorporation of both prospective and retrospective studies introduces heterogeneity, leading to variability in methodologies and data collection, which may impact the overall robustness of the conclusions.

Another limitation lies in the restricted duration of follow-up, which may impede a comprehensive assessment of the long-term outcomes associated with sphenopalatine artery ligation or cauterization. Prolonged follow-up is crucial for understanding the enduring effects of treatment and identifying potential late complications. The variability in sample sizes across the included studies, ranging from 4 to 65 participants, could influence the statistical power of the analysis, with larger sample sizes offering more reliable estimates of treatment effects.

The diverse range of risk factors for epistaxis among included patients, such as arterial hypertension, medication use, and various underlying health conditions, introduces potential confounding factors that could influence study outcomes. Additionally, the absence of prospective randomized controlled trials directly comparing sphenopalatine artery ligation and cauterization limits the ability to draw robust conclusions regarding the comparative effectiveness of these interventions.

An additional limitation of our study could be represented by the fact that this review shows a bias towards older literature, as 13 out of 16 reviewed articles are over 10 years old. This could influence the findings due to changes in surgical practices over time. The novelty of SPA ligation has diminished in the past decade, potentially leading to underrepresentation of recent advancements and optimal outcomes in the literature.

Finally, the incomplete reporting of complications in some studies and the variability in reported complications pose challenges in assessing the overall safety of sphenopalatine artery ligation or cauterization.

To improve the comparability and quality of data reported in future studies, we recommend evaluating:

- Risk Factors.
  - Patient Age: Report the mean age and distribution by age groups.
  - Comorbidities: List all relevant comorbidities, particularly cardiovascular diseases, coagulopathies, liver diseases, autoimmune diseases, alcohol and cocaine abuse, diabetes, and other chronic conditions.
  - Medication Use: Specify the use of antiplatelet, anticoagulant, and other relevant medications.
- Specific Complications.
  - Intraoperative Complications: Describe all complications that occur during the procedure (intraoperative bleeding, maxillary nerve injury, orbital injury, nasolacrimal duct injury, nasal septal perforation etc.)
  - Immediate Postoperative Complications: Report complications within the first 24–48 h post-intervention (rebleeding, decreased lacrimation, numbness of the teeth, palate or upper lip, diplopia, acute sinusitis, altered smell and taste etc.)
  - Long-term Complications: Monitor and report complications occurring after 30 days from the intervention (rebleeding, nasal septal perforation, chronic nasal dryness, atrophic rhinitis, chronic sinusitis, scarring and adhesions, chronic nasal congestion, etc.)
- Follow-Up Duration.
  - Minimum Follow-Up: Ensure a minimum follow-up of 6 months for all patients.
  - Follow-Up Visits: Plan follow-up visits at 1 month, 3 months, and 6 months post-intervention.
  - Follow-Up Methodology: Clearly describe the methodologies used for follow-up (e.g., clinical visits, phone calls, questionnaires).
- Specific Outcomes.
  - Rebleeding Rate: Report the rebleeding rate divided into early (< 30 days) and late (> 30 days) rebleeding.
  - Patient Satisfaction: Include patient satisfaction questionnaires.

By following these guidelines, greater consistency and quality in reported results can be ensured, facilitating comparison across different studies and contributing to a better understanding of the efficacy and safety of these procedures.

## Conclusions

In conclusion, our findings underscore the complexity of spontaneous epistaxis, prevalent among the elderly population with multiple risk factors, predominantly cardiovascular diseases, and antiplatelet/anticoagulant therapy.

The meticulous examination of included studies revealed a preference for the reliability and expediency of ligation as a surgical technique mainly due to its cost-effectiveness. Furthermore, the laterality of the procedure and the distinction between early and late recurrences shed light on the nuances of postoperative outcomes. The low incidence of complications, predominantly temporary decreased lacrimation in patients undergoing ligation of the SPA, emphasizes the safety and feasibility of these interventions.

In essence, our systematic review contributes valuable insights into the evolving landscape of managing intractable epistaxis, providing clinicians with evidence-based perspectives for informed decision-making in challenging cases. Future research should continue to explore innovative approaches and refine our understanding of this intricate clinical scenario.
